# Activation of AIM2 inflammasome cascade by extracellular trap-laden neutrophil vesicles triggered by *Fusobacterium nucleatum* exacerbates ulcerative colitis

**DOI:** 10.1016/j.gendis.2025.102004

**Published:** 2025-12-24

**Authors:** Linwei Xiang, Yuqing Zhao, Yaqian Duan, Jia Xu, Yan You, Xuehua Kong, Jianbo Zhang, Rui Wu, Lan Zhou, Liang Duan

**Affiliations:** a Key Laboratory of Laboratory Medical Diagnostics, Ministry of Education, Department of Laboratory Medicine, Chongqing Medical University, Chongqing 400016, China; b Department of Laboratory Medicine, The Second Affiliated Hospital of Chongqing Medical University, Chongqing 400010, China; c Department of Pathology, The Second Affiliated Hospital of Chongqing Medical University, Chongqing 400010, China; d Department of Gastrointestinal Surgery, The Second Affiliated Hospital of Chongqing Medical University, Chongqing 400010, China; e Department of Laboratory Medicine, The First Affiliated Hospital of Chongqing Medical University, Chongqing 400016, China

**Keywords:** AIM2 inflammasome, Extracellular vesicles, NETs, Pyroptosis, Ulcerative colitis

## Abstract

Neutrophil dysfunction and activation of the absent in melanoma 2 (AIM2) inflammasome have been implicated in ulcerative colitis (UC) pathogenesis. However, whether these two immune responses interact synergistically and their precise role in UC remains unclear. Here, we performed bioinformatics and clinical analyses to assess neutrophil extracellular traps (NETs) and AIM2 inflammasome cascades in UC, including their correlation. Dual immunofluorescence revealed the predominant cell types expressing AIM2. Neutrophil activation and NETs release were examined in *Fusobacterium nucleatum* (*Fn*)-stimulated cell models, while *in vitro* and *in vivo* studies investigated the effect of NETs on AIM2 inflammasome activation, UC severity, and therapeutic potential. Bioinformatics revealed co-up-regulation and significant co-expression of AIM2 inflammasome cascade-related genes and NETs-related genes in active UC. Confocal imaging showed that AIM2 primarily localized to intestinal epithelial cells in intestinal biopsies from UC patients and colon tissues from UC model mice. *Fn* infection partially promoted neutrophil activation and the release of NETs via neutrophil extracellular vesicles (nEVs). NETs were carried by nEVs and internalized by intestinal epithelial cells, subsequently triggering AIM2 inflammasome-dependent pyroptosis. *In vivo* studies demonstrated that elevated AIM2 inflammasome-related indicators triggered by *Fn*-stimulated nEVs-NETs were related to UC severity. Therapeutically, blockade of the nEVs-NETs/AIM2 inflammasome cascade using the NET-degrading agent *DNase I* or the AIM2 inhibitor A151 effectively attenuated colitis. Collectively, AIM2 inflammasome cascade activation by NETs-laden nEVs triggered by *Fn* exacerbates ulcerative colitis, highlighting the nEVs-NETs/AIM2 inflammasome cascade as a promising therapeutic target.

## Introduction

In the past few decades, the prevalence of inflammatory bowel disease has increased globally, making it a public health concern.^1^ Ulcerative colitis (UC), one of the main types of inflammatory bowel disease, is characterized by weight loss, abdominal pain, diarrhea, and bloody stools.^2^^,^^3^ The possible etiology of UC is related to genetic, environmental, and immunological factors and intestinal barrier dysfunction.^4^ Although substantial research has demonstrated that the pathogenesis of UC is strongly linked to mucosal barrier damage and immune dysregulation, the underlying mechanisms are not fully understood. At present, traditional drug treatments have a certain relieving effect on early patients with UC, while the failure rates of these drugs remain high.^5^ Despite decades of efforts in clinical research, there is no effective therapeutic strategy for UC. Therefore, there is an urgent need to elucidate the pathogenesis of UC and identify novel clinical intervention targets.

Pyroptosis is an inflammatory programmed cell death, which ultimately results in the release of numerous inflammatory factors.^6^ According to recent reports, pyroptosis may facilitate the progression of autoimmune and autoinflammatory illnesses, including kidney diseases, ischemia-reperfusion, liver fibrosis, and colon cancer.^7^, ^8^, ^9^, ^10^ Inflammasome-initiated pyroptosis also results in intestinal damage and inflammation. Past studies related to UC have mainly focused on NACHT, LRR, and PYD domain-containing protein 3 (NLRP3) inflammasome overactivation, the blockade of which has gradually become an intervention strategy in UC models.^11^ In addition to the NLRP3 inflammasome, the absent in melanoma 2 (AIM2) inflammasome is strongly up-regulated in inflammatory bowel disease.^12^ However, it remains unclear whether AIM2 inflammasome-dependent pyroptosis participates in the pathological mechanism and whether it can become a novel target for UC treatment.

Neutrophil extracellular traps (NETs) are a unique neutrophil killing mechanism, comprising enzymes and intracellular granules that form 25-nm globular domains and aggregate into 50-nm threads.^13^^,^^14^ NETs have been implicated as mediators of pathology in sterile inflammation, such as sepsis, diabetes, and rheumatoid arthritis.^15^, ^16^, ^17^ In UC, neutrophil infiltration into the intestinal mucosa and the production of abundant NETs, which are involved in inflammatory immune disorders and intestinal epithelial barrier damage, have been reported to occur in the pathophysiology of UC.^18^^,^^19^ Nevertheless, the mechanisms remain poorly understood. In conditions such as sepsis and endometritis, NETs have been shown to trigger pyroptosis, thereby accelerating inflammation through activation of inflammasomes by NET-associated components.^20^^,^^21^ Recent evidence has further demonstrated that NETs activate AIM2 inflammasome-dependent pyroptosis in lipopolysaccharide (LPS)-induced acute respiratory distress syndrome.^22^ Consistently, our previous work confirmed that NETs promoted liver inflammation and fibrosis progression by inducing AIM2 inflammasome-dependent pyroptosis in macrophages.^23^ Notably, our previous work demonstrated that colonization of oral-derived *Fusobacterium nucleatum* (*Fn*) in the intestine stimulated NET formation and consequently contributed to colorectal cancer progression.^24^ Moreover, *Fn* has also been implicated as a key bacterium in UC pathogenesis^25^; however, whether *Fn*-induced NETs formation in neutrophils similarly contributes to UC progression remains unclear. Given the high expression of AIM2 in UC and the established role of NETs in triggering AIM2 inflammasome activation, as well as the ability of NETs to be modulated by *Fn*, we hypothesize that NETs induced by *Fn* may create a proinflammatory microenvironment by activating AIM2 inflammasome, thereby promoting UC progression.

In the current study, we examined indicators of NETs and AIM2 inflammasome-dependent pyroptosis in clinical samples and mouse models and analyzed their clinical correlation to UC severity. We also investigated the mechanisms by which they communicate with each other and their ultimate impact on the pathological process of UC. The present study demonstrated that neutrophil extracellular vesicles (nEVs) carrying the extracellular traps triggered by *Fn* were internalized by intestinal epithelial cells (IECs), thereby triggering AIM2 inflammasome-dependent pyroptosis and aggravating colitis. Targeting the nEVs-NETs/AIM2 inflammasome axis may represent a promising therapeutic strategy for UC.

## Materials and methods

### Human samples

All experiments involving human samples were conducted according to the ethical policies and procedures approved by the Ethical Committee of the First Hospital affiliated with Chongqing Medical University (Approval no. K2023-305). The peripheral blood samples from 25 patients with active UC and 23 healthy controls (HCs) were collected between July 2023 and November 2024 at the First Affiliated Hospital of Chongqing Medical University. The serum was obtained by centrifuging the peripheral blood of UC patients and HCs for 10 min and kept at −80 °C for further examination. The remaining peripheral blood samples were used to obtain neutrophils. Additionally, biopsy UC tissue specimens were also obtained from 25 patients with UC who had undergone enteroscopy, and normal tissue biopsies were collected from 5 HCs who had undergone an enteroscopy for routine colon cancer screening. Clinical disease activity was assessed using the Mayo Clinic Score (MCS), which includes an endoscopic subscore and a rectal bleeding subscore.^26^^,^^27^ Informed written consent was obtained from all participants. Patient characteristics are shown in Table 1.

**Table 1:** The clinical characteristics of enrolled individuals in this study.

Chararcteristics	Serum specimen		Tissue specimen	
	HC (*n* = 23)	UC (*n* = 25)	HC (*n* = 5)	UC (*n* = 25)
*Gender*				
Male (*n*, %)	12 (52.2%)	12 (48.0%)	1 (20.0%)	12 (48.0%)
Female (*n*, %)	11 (47.8%)	13 (52.0%)	4 (80.0%)	13 (52.0%)
*Age*				
<40 (*n*, %)	9 (39.1%)	14 (56.0%)	2 (40.0%)	14 (56.0%)
≥40 (*n*, %)	14 (60.9%)	11 (44.0%)	3 (60.0%)	11 (44.0%)
*Location*				
Rectum (*n*, %)	NA	5 (20.0%)	NA	5 (20.0%)
Left side (*n*, %)	NA	11 (44.0%)	NA	11 (44.0%)
Totalcolitis (*n*, %)	NA	9 (36.0%)	NA	9 (36.0%)
*Mayo Clinic score*				
Mild (*n*, %)	NA	12 (48.0%)	NA	12 (48.0%)
Moderate/severe (*n*, %)	NA	13 (52.0%)	NA	13 (52.0%)

Note: HC, healthy control; NA, not applicable; *n*, number of samples; UC, ulcerative colitis.

### Cell culture

The human normal colon epithelial cell line NCM-460 was preserved at the Key Laboratory of Laboratory Medical Diagnostics, Ministry of Education, Chongqing Medical University (Chongqing, China) and was used in our previous studies.^28^ The NCM-460 cells were cultured in Dulbecco’s modified Eagle’s medium (DMEM, Gibco, USA) and incubated at 37 °C with 5% CO_2_ and 95% air. The medium contained 10% fetal bovine serum (Clark Bioscience, USA) and 1% penicillin/streptomycin (HyClone, USA).

### Bacterial strains

The *Fn* strain ATCC 25586 was purchased from the American Type Culture Collection (ATCC, Manassas, Virginia, USA). As previously described, the bacteria were cultured in brain heart infusion.^24^ *Escherichia coli* (*E. coli*) (DH5α, Tiangen, China) was cultured for 24 h in Luria–Bertani (LB) medium at 37 °C overnight in an orbital shaker incubator. Both bacteria were collected through centrifugation at 2500 rpm for 10 min and then washed using phosphate-buffered saline. After that, the concentration of the bacteria was adjusted to 1 × 10^9^ colony-forming units per milliliter. All subsequent experiments were conducted with a multiplicity of infection (MOI) of 100:1.

### AIM2 gene knockdown

The *AIM2* gene was knocked down using siRNA (GenePharma, China) in accordance with the manufacturer’s instructions. The siRNA sequences and quantitative PCR primers are shown in Table S1.

### RNA extraction and quantitative PCR

Total cellular RNA was obtained with Trizol reagents (Invitrogen, USA). cDNA was reverse-transcribed with a reverse transcription kit (Takara, Japan). The mRNA levels of AIM2 were analyzed with the CFX96 real-time PCR detection system (Bio-Rad, USA) using SYBR Green dye (Biomake, China). GAPDH served as a reference control. The –ΔCT method was utilized to compute the fold changes in gene expression. The primer pairs used are shown in Table S1.

### Human neutrophil isolation and NET formation assay

Neutrophils were separated from UC and HC using a human peripheral blood neutrophil isolation kit (P9040, Solarbio, China) according to the manufacturer’s instructions. Briefly, neutrophil-separating medium and 4 mL fresh anticoagulant whole blood were added to a 15 mL tube in sequence, and centrifuged at 1000 *g* at room temperature for 40 min. The neutrophil-rich band was collected into another fresh 15 mL tube. After erythrocyte lysis and a series of washes with phosphate-buffered saline, isolated neutrophils were resuspended in RPMI 1640 without fetal bovine serum.

The assay for NET formation was carried out as previously described.^29^ After being stimulated with Phorbol 12-myristate 13-acetate (PMA, 100 ng/mL, Sigma, USA) and incubated at 37 °C for 4 h, neutrophils and bottom released NETs were obtained by centrifugation at 4 °C at 450 *g* for 10 min. Then, NET-rich supernatant was collected after centrifugation at 4 °C at 18000 *g* for 10 min. After discarding the supernatant, NET suspension was prepared in ice-cold phosphate-buffered saline. NETs-DNA concentration was measured by NanoDrop spectrophotometer (ThermoFisher, USA).

### Isolation of nEVs from cultured cell conditioned medium

Isolated neutrophils were treated with LPS (2 μg/mL, L2630, Sigma–Aldrich, USA) or *Fn* (multiplicity of infection of 100:1), with or without Deoxyribonuclease I (*DNase I*, 100 U/mL, a NETs-degrading agent), at 37 °C for 20 h. The supernatant was then collected to isolate neutrophil extracellular vesicles (nEVs) using an extracellular vesicle isolation kit (BB-4564-50T, BsetBio, China) in accordance with the manufacturer’s guidelines. Briefly, the obtained neutrophil culture medium was subjected to ultra-high-speed centrifugation (4 °C, 10,000 *g*, 20 min) to collect the supernatant. The supernatant was then filtered using a kit-specific centrifuge tube, followed by the addition of vesicle extraction solution to obtain a purified vesicle suspension. The size distribution and particle concentration of isolated nEVs were analyzed using nanoparticle tracking analysis. Measurements were performed with a ZetaView® PMX 120 instrument (Particle Metrix, Germany) and processed using ZetaView software (v 8.05.12). For nEV characterization, nEVs were lysed in RIPA buffer and analyzed by Western blotting for EV markers (CD63, HSP70, Annexin V, Alix, and Calnexin). The isolated nEVs from neutrophils stimulated with LPS or *Fn* were subsequently used for *in vitro* and *in vivo* experiments. In addition, the nEVs were pretreated with 0.01% Triton X-100 on ice for 5 min to release their contents, followed by ELISA analysis of MPO-DNA.

### Transmission electron microscopy

nEVs treated with or without immunogold-labeled anti-CitH3 antibodies were analyzed by transmission electron microscopy using negative staining technology. Briefly, 10 μL of a sample was dropped onto a copper mesh, and excess liquid was absorbed by filter paper 10 min later. Then 10 μL of 2% uranium acetate was dropped onto the copper mesh, and excess liquid was absorbed by filter paper after 1–3 min. After drying for 5 min, the samples were observed using a transmission electron microscope (JEM-1200EX, Japan).

### Fluorescence *in situ* hybridization analysis

*Fn* in human tissue was detected using the *Fn*-targeted probe, FUS664 (FITC-labeled), 5′-CTT GTA GTT CCG C(C/T) TAC CTC-3′. The fluorescence *in situ* hybridization analysis was performed according to the protocol previously described.^24^

### Immunohistochemistry

Briefly, tissue sections encased in paraffin were dried, deparaffinized, and heated for 10 min in a pressure pot, followed by treatment with 0.3% H_2_O_2_. Then the sections were incubated with anti-AIM2 (1:200, ab93015, Abcam, UK), anti-IL-1β (1:200, AF02479, AiFang biological, China), and anti-GSDMD (1:200, 20770-1-AP, Proteintech, China), followed by secondary antibody incubation labeled with the peroxidase enzyme (1:200, SP-9001, Zhongshan Golden Bridge, China) for 30 min. After counterstaining the slides with hematoxylin and visualizing them with 0.05% diaminobenzidine, the slides were examined under a Nikon E400 light microscope. The mean of integrated optical density for immunohistochemistry-stained materials was analyzed using the Image Pro Plus (v 6.0.0.260).

### Immunofluorescence

NCM-460 cells or neutrophils subjected to various treatments were seeded on a climbing piece. After fixation and permeabilization, cells were blocked with 10% goat serum for 1 h. Neutrophils were incubated with anti-citrullinated modification of histone 3 (CitH3) antibody (1:1000, ab5103, Abcam, UK), anti-myeloperoxidase (MPO) antibody (1:1000, ab208670, Abcam, UK), and anti-neutrophil elastase (NE) antibody (1:200, 89241, CST, USA) at 4 °C overnight. The following day, cells were incubated with Alexa Fluor 594-conjugated goat anti-rabbit secondary antibody (1:200, SA00013-4, Proteintech, China) in the dark for 1 h. The cell cytoskeleton of NCM-460 cells was stained by YF® 488-Phalloidin (1:100, YP0059S, UE) for 15 min, and the nucleus was counterstained with DAPI (MBB039, MengBio, China) for 10 min. After sealing the slide, images were examined under the confocal microscope (Leica, Germany).

Tissue sections were subjected to deparaffinization, rehydration, and antigen retrieval. The sections or cells with various treatments were incubated with primary antibodies: anti-AIM2 (1:200, ab93015, UK), anti-Ly6G (1:100, 14-5931-81, Invitrogen, USA), anti-CD66b (1:200, ab197678, Abcam, UK), anti-CD68 (1:400, 97778, CST, USA), anti-F4/80 (1:500, 70076, CST, USA), anti-CitH3 (1:1000, ab5103, Abcam, UK), and anti-E-cadherin (1:300, 20874-1-AP, Proteintech, China) overnight, followed by secondary antibody incubation in a dark environment for 1 h. Finally, images were acquired using a confocal microscope (Leica, Germany).

### Western blotting

Equal amounts of protein (30 μg) were subjected to sodium dodecyl sulfate-polyacrylamide gel electrophoresis (SDS-PAGE) and transferred to polyvinylidene difluoride (PVDF) membranes. The membranes were incubated with primary antibodies at 4 °C overnight after blocking with 5% bovine serum albumin for 2 h. Primary antibodies included anti-CitH3 (1:1000, ab5103, Abcam, UK), anti-AIM2 (1:1000, ab93015, Abcam, UK), anti-GSDMD (1:5000, 20770-1-AP, Proteintech, China), anti-cleaved caspase-1 (1:1000, 4199T, CST, USA), anti-cleaved-IL-1β (1:1000, 83186, CST, USA), anti-MPO antibody (66177-1-Ig, Proteintech, China), anti-NE antibody (1:1000, 89241, CST, USA), anti-HSP70 (1:1000, M05211019, Wanleibio, China), anti-CD63 (1:1500, M04122549, Wanleibio, China), anti-Annexin V (1:500, M11300006, Wanleibio, China), anti-Alix (1:2000, M10293063, Wanleibio, China), anti-Calnexin antibody (1:5000, 10427-2-AP, Proteintech, China), and anti-β-actin (1:10000, 66009-1-Ig, Proteintech, China). Then, horseradish-peroxidase-conjugated secondary antibodies were used to incubate the membranes at 37 °C for 1 h. The peroxidase activity of secondary antibodies was tested with ECL Western blot substrate and recorded by Bio-Rad Electrophoresis Documentation (ECL, Millipore, Germany). All the experiments were performed three times. Protein band intensities were analyzed using Image J software (v1.49p, Java 1.6.0_20).

### ELISA

Proteins of interest in serum or cell supernatant were tested using ELISA in compliance with the manufacturer’s guidelines. ELISA kits were as follows: mouse TNF-α (RX202412M, RuiXin Biotech, China), mouse IL-6 (RX203049M, RuiXin Biotech, China), mouse IL-1β (RX203063M, RuiXin Biotech, China), mouse AIM2 (RX203426, RuiXin Biotech, China), mouse IL-18 (RX203064M, RuiXin Biotech, China), mouse GSDMD (RX200648M, RuiXin Biotech, China), human AIM2 (MBE8322, mengbio, China), human GSDMD (MBE1217, mengbio, China), human IL-1β (MBE0058, mengbio, China), human IL-18 (MBE0066, mengbio, China), and human Fap2 (JM-6778H2, jingmeibio, China).

MPO–DNA complexes were assessed via capture ELISA using a commercial cell death detection ELISA kit (11774425001, Roche, Germany). 20 μg of anti-MPO antibody (700 μg/mL, 66177-1-Ig, Proteintech, China) was coated onto 96-well microtiter plates at 4 °C overnight. Then, 20 μL serum or nEVs lysate was added and shaken at 320 rpm for 2 h after blocking with 1% bovine serum albumin. After three washes with 250 μL of incubation buffer per well, 100 μL peroxidase substrate (ABTS) was added and shaken at 200 rpm in the dark for 15 min. The absorbance at 405 nm was measured using a microplate reader.

### Flow cytometry

NCM-460 cells were treated with 2 μg/mL NETs for 24 h or co-cultured with neutrophils pre-treated with LPS (1 μg/mL), LPS + *DNase I* (100 U/mL), or LPS + GW4869 (2 μg/mL, HY-19363, MCE, USA) for 48 h. FLICA 660-YVAD-FMK (FLICA 660 *in vitro* active Caspase-1 detection kit; ImmunoChemistry Technologies, USA) and propidium iodide (PI, Life Technologies, USA) were used to detect pyroptotic death in cells. All the experiments were performed three times. The mean fluorescence intensity was quantified using FlowJo software (v 10.8.1).

### EVs fluorescence labelling

The nEVs were labeled with fluorescent lipophilic tracer Dil (2 μg/mL, C1036, Beyotime, China). Briefly, labeling was performed for 5 min at 37 °C and then 15 min at 4 °C in the dark. To get rid of any leftover free dye, labeled EVs were centrifuged again according to the extracellular vesicle isolation kit manufacturer’s protocols. The labeled EVs were used to treat NCM-460, and cellular uptake was assessed using a fluorescence microscope.

### Induction of colitis

All experiments involving animals were conducted according to the ethical policies and procedures approved by the Animal Ethics Committee of Chongqing Medical University (Approval no. 2022141). Male C57BL/6 mice (20–22 g, 8 weeks old) were purchased from Chongqing Tengxin Biotechnology Limited Company and housed under standard laboratory conditions.

Mice (*n* = 8 per group) were randomly divided into control and DSS groups. Acute colitis was induced through administration of 3% dextran sodium sulphate (DSS, molecular mass, 36,000–50,000 Da, Yeansen Biotech, China), dissolved in autoclaved drinking water, for 8 days. Body weight, fecal morphology, and the presence of gross blood in feces and anus were all monitored daily. After 8 days, the cardiac puncture was used to get the blood samples, and serum was obtained and stored at −80 °C following centrifugation at 4000 rpm for 15 min. The spleen was separated to obtain the spleen index (spleen weight/mouse weight). Colon length was measured from the cecum to the anal verge. The distal colon was taken and fixed in 4% paraformaldehyde for immunohistochemistry and hematoxylin-eosin staining. The histology score was blindly determined based on damaged area, mucodepletion of gland, tissue damage, and inflammatory cell infiltration.^30^ The sum of the scores from each category was used to calculate the overall histopathological score.

C57BL/6 mice (*n* = 40) underwent the same DSS protocol for serum collection. ELISA was performed to quantify MPO-DNA and AIM2 inflammasome-dependent pyroptosis-related indicators AIM2, GSDMD, and IL-18.

C57BL/6 (*n* = 20) mice were randomly split into five groups: control + saline; DSS + saline; DSS + *Fn*-stimulated nEVs (*Fn*-nEVs); DSS + *Fn*-nEVs + *DNase I* (SLBV9316, Sigma, USA) (*Fn*-nEVs + *DNase I*); DSS + A151 (HY-150751, MCE, USA; an AIM2 inflammasome inhibitor). Consistent with the previous experimental methods, colitis was induced by administering 3% DSS dissolved in water for 8 days. During the molding process, mice in the DSS + *Fn*-nEVs group and the DSS + *Fn*-nEVs + *DNase I* group were additionally given *Fn*-nEVs (50 μg/each mouse) or *Fn*-nEVs pretreated with *DNase I* (50 μg/each mouse) via intraperitoneal injection once every other day. *Fn*-nEVs were isolated and *DNase I*-treated as described in the *in vitro* experiments. Meanwhile, mice in the DSS + A151 group were administered intraperitoneal injections of A151 (100 μg/each mouse) once every two days. All mice were euthanized on day 9, and tissues were processed as above.

### Statistical analysis

The dataset (GSE59071) was downloaded from the GEO database (http://www.ncbi.nlm.nih.gov/geo/). Bioinformatics analysis of NETs-related genes^31^ and genes of AIM2-dependent pyroptosis^32^ was performed using RStudio (version 4.2.2). All *in vitro* experiments were performed in three independent experiments. Data were expressed as mean ± standard deviation or as median (interquartile range, IQR), and were statistically analyzed using GraphPad Prism software (v10.1.2). Serum levels of AIM2, GSDMD, IL-1β, IL-18, and MPO-DNA were analyzed using the Kruskal–Wallis or Mann–Whitney *U* test. Spearman correlation was used to calculate correlation coefficients (*r*). For cell-based experiments, Student’s *t*-test (two groups) or one-way ANOVA with Newman–Keuls’ multiple comparison test (≥ 3 groups) was used to determine significance. ∗*P* < 0.05, ∗∗*P* < 0.01, and ∗∗∗*P* < 0.001 were considered statistically significant.

## Results

### AIM2 and its downstream molecules are elevated in UC

The dataset (GSE59071), containing HCs (*n* = 11), inactive UC (*n* = 23), and active UC (*n* = 74), was downloaded from the GEO database. The heatmap analysis showed that genes of AIM2 and its downstream pyroptosis-related molecules, including *AIM2*, *caspase-1*, *GSDMD*, *IL-18*, and *IL-1β*, exhibited distinct expression patterns among different groups, and the results indicated that the above genes were strongly up-regulated in active UC (Fig. 1A). Significant differences in these genes expression were also observed between HC and UC (Fig. 1B). Based on the bioinformatic data, we then collected clinical biopsy tissues and serum samples from UC for further validation as described above. Hematoxylin-eosin staining confirmed that the patients with UC had extensive tissue damage, as indicated by the existence of large areas of tissue disorder with crypt loss and inflammatory cell infiltration (Fig. 1C). Immunohistochemistry staining revealed increased expression of AIM2, IL-1β, and GSDMD in biopsy tissues from patients with UC compared with HCs (Fig. 1C). ELISA data also showed increased levels of serum AIM2, GSDMD, IL-1β, and IL-18 in patients with UC (Fig. 1D–G). To further investigate which cell types predominantly exhibit elevated AIM2, we examined its colocalization with the neutrophil marker CD66b, the macrophage marker CD68, and the IEC marker E-cadherin in biopsy tissues. AIM2 expression was significantly higher in UC patients and mostly localized to IECs in intestinal biopsy tissues from the patients with UC (Fig. 1H). The above results were also validated in a 3% DSS-induced UC mouse model (Fig. 1I). Successful establishment of the UC model was confirmed by colon shortening and tissue damage with inflammatory cell infiltration and increased serum proinflammatory factor (TNF-α, IL-6, and IL-1β) release (Fig. 1J–N). Body weight loss, disease activity index, spleen index, and histological score were also used to verify the successful establishment of this model (Fig. S1A–S1D). Consistent with the human data, the expression of AIM2, GSDMD, and IL-18 was also increased in the colon of the DSS-induced UC model mice (Fig. 1O–Q). As predicted, confocal images of mouse colonic sections showed that IECs were the primary cell type with elevated AIM2 expression (Fig. 1R).

**Figure 1:**
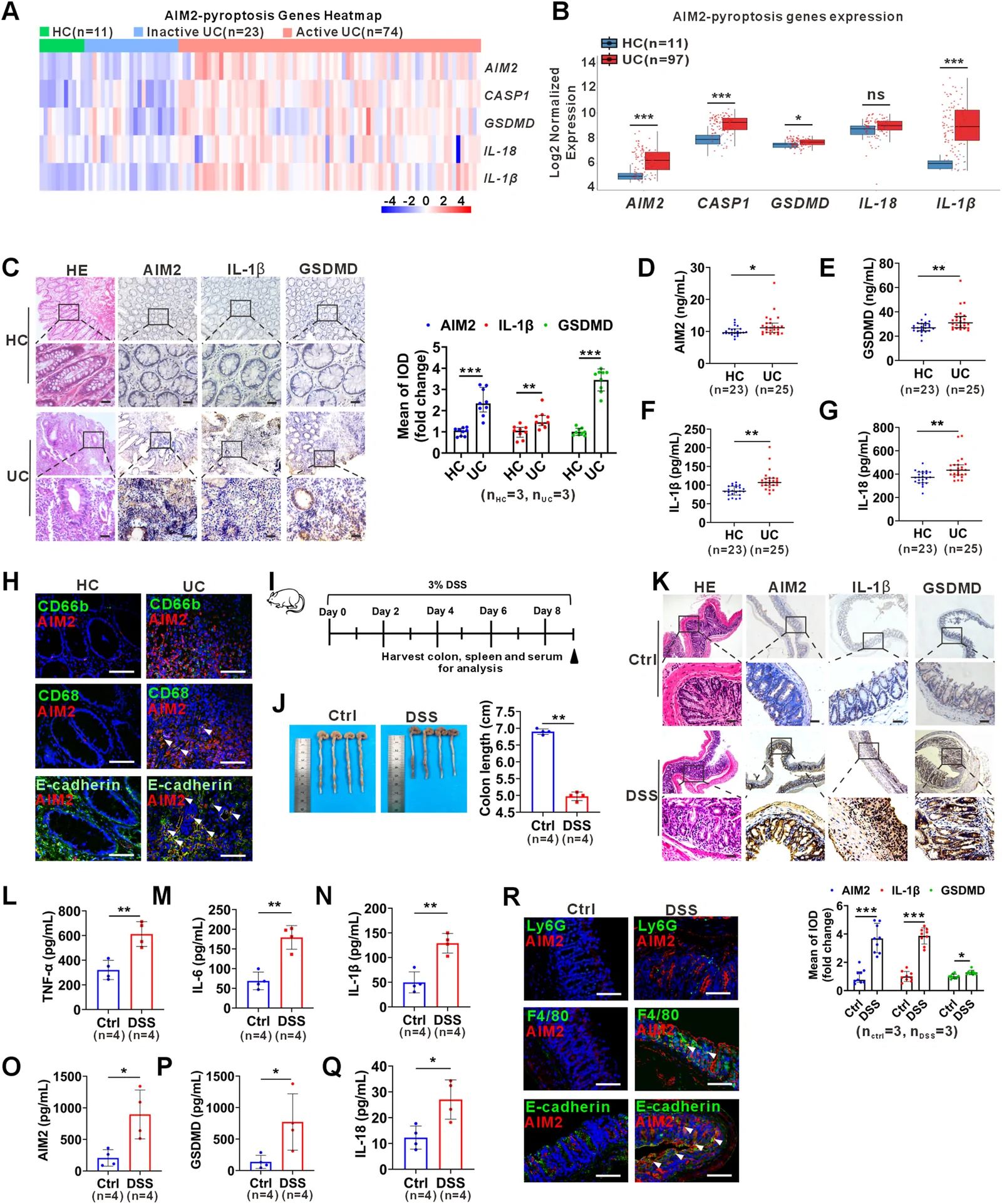
AIM2 and its downstream pyroptosis-related molecules are elevated in ulcerative colitis (UC). **(A)** Analysis of genes of *AIM2* and its downstream pyroptosis-related molecules in healthy control (HC), inactive UC, and active UC from the GEO database (GSE59071). A heatmap showing differences in the distribution of *AIM2*-pyroptosis genes among different groups. **(B)** Boxplot showing differences in the expression of *AIM2*, *caspase-1*, *GSDMD*, *IL-18*, and *IL-1β*. **(C)** Representative human colon histology of hematoxylin-eosin staining and immunohistochemistry staining for AIM2, IL-1β, and GSDMD from patients with UC and HCs. The mean of integrated optical density (IOD) for immunohistochemistry-stained materials was analyzed utilizing the Image Pro Plus software, and data from histomorphometric analysis were calculated from 3 random fields of each tissue section from HCs (*n* = 3) and patients with UC (*n* = 3). **(D**–**G)** ELISA analysis of serum levels of AIM2 (D), GSDMD (E), IL-1β (F), and IL-18 (G) in HCs (*n* = 23) and patients with UC (*n* = 25). **(H)** Representative immunofluorescence images of AIM2 (red) and CD66b (human neutrophil marker), CD68 (human macrophage marker), or E-cadherin (intestinal epithelial cell marker) (green) from biopsy tissues of UC. **(I)** Schematic diagram of the study. Acute colitis was induced through the administration of 3% dextran sulfate sodium (DSS), dissolved in autoclaved drinking water, for 8 days. **(J)** Representative pictures for the length of the colon and statistical analysis in the control (Ctrl) and DSS groups. **(K)** Representative mouse colon histology of hematoxylin-eosin staining and immunohistochemistry staining for AIM2, IL-1β, and GSDMD. The mean of IOD for immunohistochemistry-stained materials was analyzed utilizing the Image Pro Plus software, and data from histomorphometric analysis were calculated from 3 random fields of each tissue section from 3 randomly selected mice in each group. **(L**–**N)** ELISA analyses of serum levels of TNF-α (L), IL-6 (M), and IL-1β (N) in DSS group mice (*n* = 4) and Ctrl group mice (*n* = 4). **(O**–**Q)** ELISA analyses for serum levels of AIM2 (O), GSDMD (P), and IL-18 (Q) in DSS group mice (*n* = 4) and Ctrl group mice (*n* = 4). **(R)** Representative immunofluorescence images of AIM2 and Ly6G (mouse neutrophil marker), F4/80 (mouse macrophage marker), or E-cadherin in tissues from the 8-day 3% DSS-treated mouse. Statistical results in panels (D–G) were presented as median (IQR), while other data were presented as mean ± standard deviation. Comparisons in panels (D–G) were performed using the Mann–Whitney *U* test, whereas comparisons in the other panels were performed using Student’s *t*-test. ns, not significant; ∗*P* < 0.05, ∗∗*P* < 0.01, and ∗∗∗*P* < 0.001. Black scale bars: 100 μm. White scale bars: 200 μm.

### NET levels are elevated in UC

The dataset (GSE59071) was further explored to analyze the expression of NET-related genes in UC. The heatmap analysis demonstrated distinct gene expression pattern differences among different groups (including HC, inactive UC, active UC) in NET-related genes, which were strongly up-regulated in active UC (Fig. 2A). The expression of the above-mentioned genes also exhibited differences between HCs and those with UC (Fig. 2B). Based on the bioinformatic data, we then analyzed NET levels in clinical biopsy tissue and serum samples from UC. CitH3, a specific marker for NETs, was analyzed by immunofluorescence staining to identify NET production. Notably, NET levels were increased in tissues of patients with UC (Fig. 2C). Furthermore, MPO-DNA, a serum marker of circulating NETs, was shown to be considerably higher in the serum of patients with UC (Fig. 2D). This finding was further supported by the increase in CitH3 in freshly isolated circulating neutrophils from the patients with UC (Fig. 2E and F). Here, we constructed another set of identical animal models to harvest serum and colon tissues (Fig. 2G). Consistent with the human data, the DSS-induced UC model also showed elevated NET levels in colonic tissues and increased MPO-DNA levels in the serum (Fig. 2H and I).

**Figure 2:**
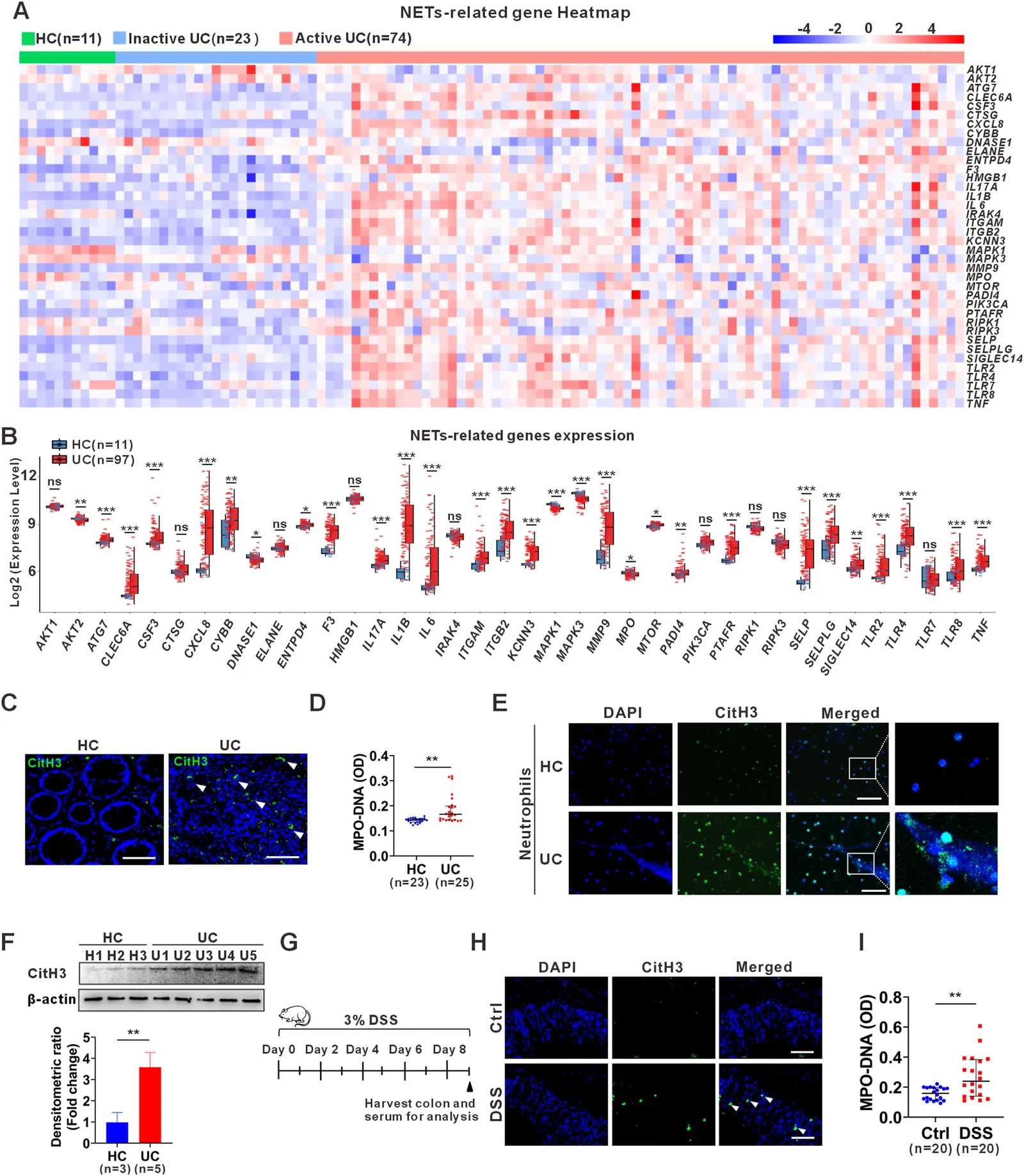
Neutrophil extracellular trap (NET) levels are elevated in ulcerative colitis (UC). **(A)** Analysis of NET-related genes in healthy control (HC), inactive UC, and active UC from the GEO database (GSE59071). A heatmap showing differences in the distribution of NETs-related genes among different groups. **(B)** Boxplot showing differences in the expression of NET-related genes. **(C)** Representative immunofluorescence images of CitH3 (green) from biopsy tissues of UC. **(D)** ELISA analysis for MPO-DNA levels in the serum from HCs (*n* = 23) and patients with UC (*n* = 25). **(E)** Representative immunofluorescence images of CitH3 in neutrophils of peripheral blood from patients with UC and HCs. **(F)** Western blotting analysis of CitH3 expression in freshly isolated neutrophils from HCs (*n* = 3) and patients with UC (*n* = 5). CitH3 protein levels were quantified using densitometry and normalized to β-actin and are shown as fold changes compared with the HC. **(G)** Schematic diagram of the study. **(H)** Representative immunofluorescence images of CitH3 (green) from the 8-day 3% dextran sulfate sodium (DSS)-treated mouse colon tissue. **(I)** ELISA analysis for MPO-DNA levels in the serum from the DSS-induced UC group (*n* = 20) and the control (Ctrl) group (*n* = 20). Statistical results in panels (D, I) were presented as median (IQR), while other data were presented as mean ± standard deviation. Comparisons in panels (D, I) were performed using the Mann–Whitney *U* test, whereas comparisons in panel F were performed using Student’s *t*-test. ns, not significant; ∗*P* < 0.05, ∗∗*P* < 0.01, and ∗∗∗*P* < 0.001. White Scale bar: 200 μm. CitH3, citrullinated modification of histone 3.

### NETs are positively correlated with the AIM2 inflammasome cascade, and both are associated with UC severity

We next analyzed the differential expression of NET-related genes and AIM2 and its downstream pyroptosis molecules-related genes in active and inactive UC. The analysis results based on the database (GSE59071) showed that both groups of genes were generally up-regulated in active UC (Fig. 3A, B). In addition, the heatmap analysis showed that genes of AIM2 and its downstream pyroptosis-related molecules in UC were positively correlated with NET-related genes, especially *AIM2*, *IL-1β*, *caspase-1*, and *GSDMD* (Fig. 3C). Since both genes of NETs and AIM2 inflammasome cascades were linked with active UC, we further validated their correlation in the biopsy tissue and serum samples from patients with active UC. The disease severity of patients with UC was assessed by the MCS. Patients with moderate/severe UC expressed more CitH3 and AIM2, as well as downstream pyroptosis-related proteins, than those with mild UC (Fig. 3D). Moreover, serum NET marker MPO-DNA and AIM2 downstream indicators IL-1β, GSDMD, and IL-18 were markedly greater in patients with moderate/severe UC (Fig. 3E–H). Given the analogous tendency with respect to the degree of UC, we next assessed the correlation of serum MPO-DNA concentrations with AIM2 expression and downstream indicators. The serum MPO-DNA concentration was found to be positively correlated with the expression of these pyroptosis-related indicators (Fig. 3I–L). A similar phenomenon was also verified in the UC mouse model, which was divided into mild and moderate/severe groups according to the histological score. The data showed that the moderate/severe group expressed more NETs and the AIM2 and its downstream pyroptosis-related proteins in tissues and serum than the mild group (Fig. S2A–S2E). As expected, a positive correlation was observed between the serum MPO-DNA concentration and AIM2 and its downstream pyroptosis-related indicators in the UC mouse model (Fig. S2F–S2H).

**Figure 3:**
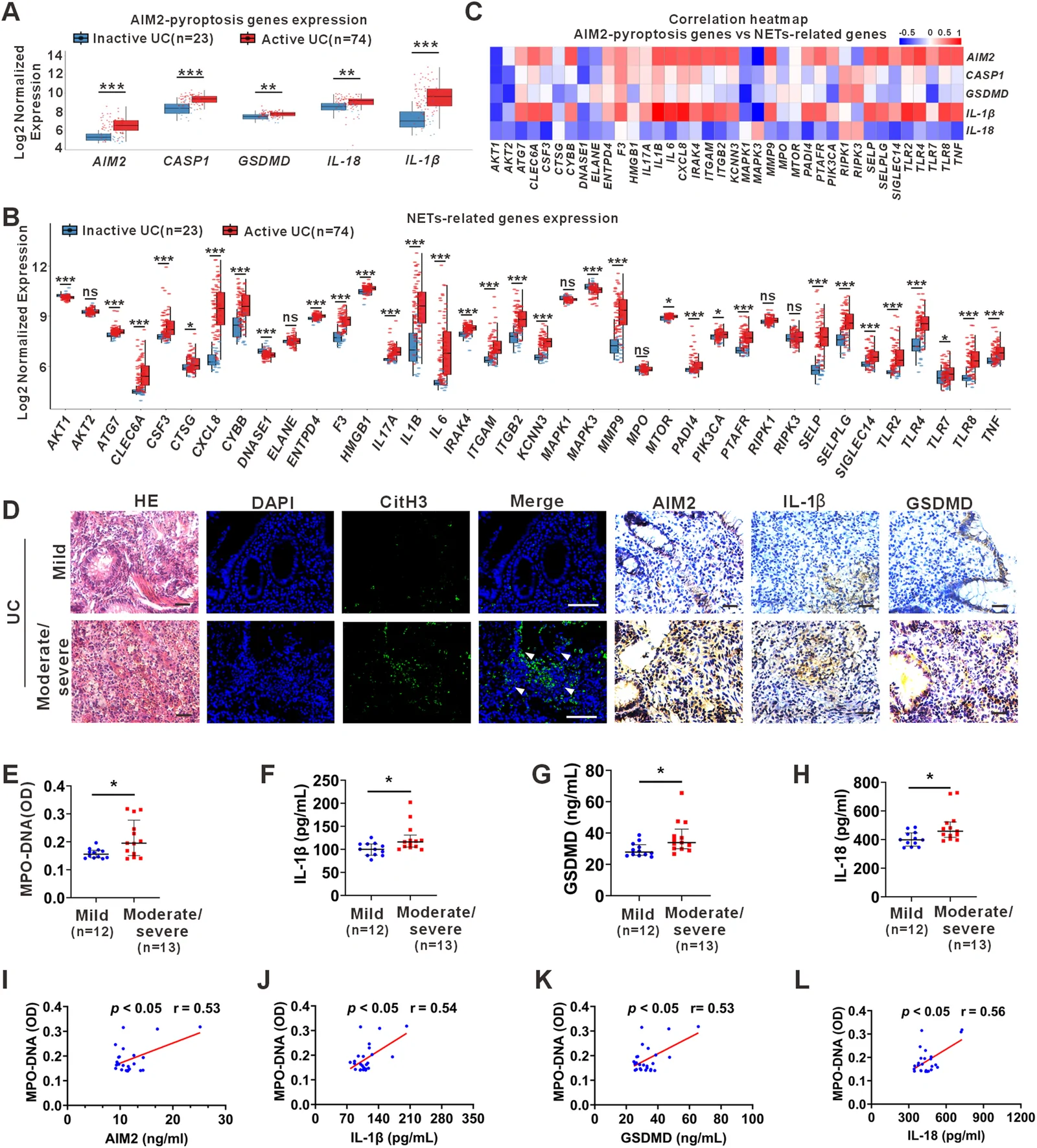
Neutrophil extracellular traps (NETs) are positively correlated with the AIM2 inflammasome cascade, and both are associated with ulcerative colitis (UC) severity. **(A)** Analysis of *AIM2*-pyroptosis gene expression in inactive UC and active UC from the GEO database (GSE59071). Boxplot showing differences in the expression of *AIM2*, *caspase-1*, *GSDMD*, *IL-18*, and *IL-1β*. **(B)** Boxplot showing differences in the expression of NET-related genes. **(C)** A heatmap showing the correlation between the *AIM2*-pyroptosis genes and NET-related genes in UC. **(D)** Representative human colon histology of hematoxylin-eosin, immunofluorescence images of CitH3 (green), and immunohistochemistry staining for AIM2, IL-1β, and GSDMD from patients with UC. **(E**–**H)** ELISA analysis for MPO-DNA (E), IL-1β (F), GSDMD (G), and IL-18 (H) levels in the serum from patients with mild (*n* = 12) and moderate/severe (*n* = 13) UC. **(I**–**L)** Correlation between serum levels of MPO-DNA and AIM2 (I), IL-1β (J), GSDMD (K), or IL-18 (L) in patients with UC. The statistical results were presented as median (IQR). Differences between mild and moderate/severe groups were tested by the Mann–Whitney *U* test. ns, not significant; ∗*P* < 0.05, ∗∗*P* < 0.01, and ∗∗∗*P* < 0.001. Black scale bars: 100 μm; White scale bars: 200 μm. CitH3, citrullinated modification of histone 3.

### NET-laden EVs from *Fn*-stimulated neutrophils are linked to AIM2-dependent pyroptosis in IECs

Fluorescence *in situ* hybridization and immunofluorescence were performed to evaluate the enrichment of *Fn* and NET levels in biopsy tissues from patients with UC. In patients with moderate/severe UC, the expression of NETs and the enrichment of *Fn* were higher than those in patients with mild UC (Fig. 4A). Serum MPO-DNA levels (NET biomarker) positively correlated with Fap2, a virulence factor secreted by *Fn* (Fig. 4B). Given the above results, we speculated that enhanced NET levels may be related to *Fn* enrichment in UC, which was verified *in vitro*. Neutrophils stimulated with *Fn* (LPS stimulation as a positive control) showed obvious web-like NET formation, visualized by morphological staining of MPO/NE (Fig. 4C). Similar trends were further verified by analysis of CitH3 expression in neutrophils (Fig. 4D). Additionally, *E. coli* was used as the control bacterium to conduct the above experiments, and no NET induction was observed with *E. coli* (Fig. S3A and S3B). Then, nEVs released after *Fn* stimulation were extracted and characterized by morphology, size, distribution, and EV markers, which showed that *Fn* stimulation resulted in the release of nEVs (Fig. 4E–G). nEVs released after *Fn* stimulation contained NET components, CitH3, MPO, and MPO-DNA, similar to those observed after LPS stimulation (Fig. 4H–J).

**Figure 4:**
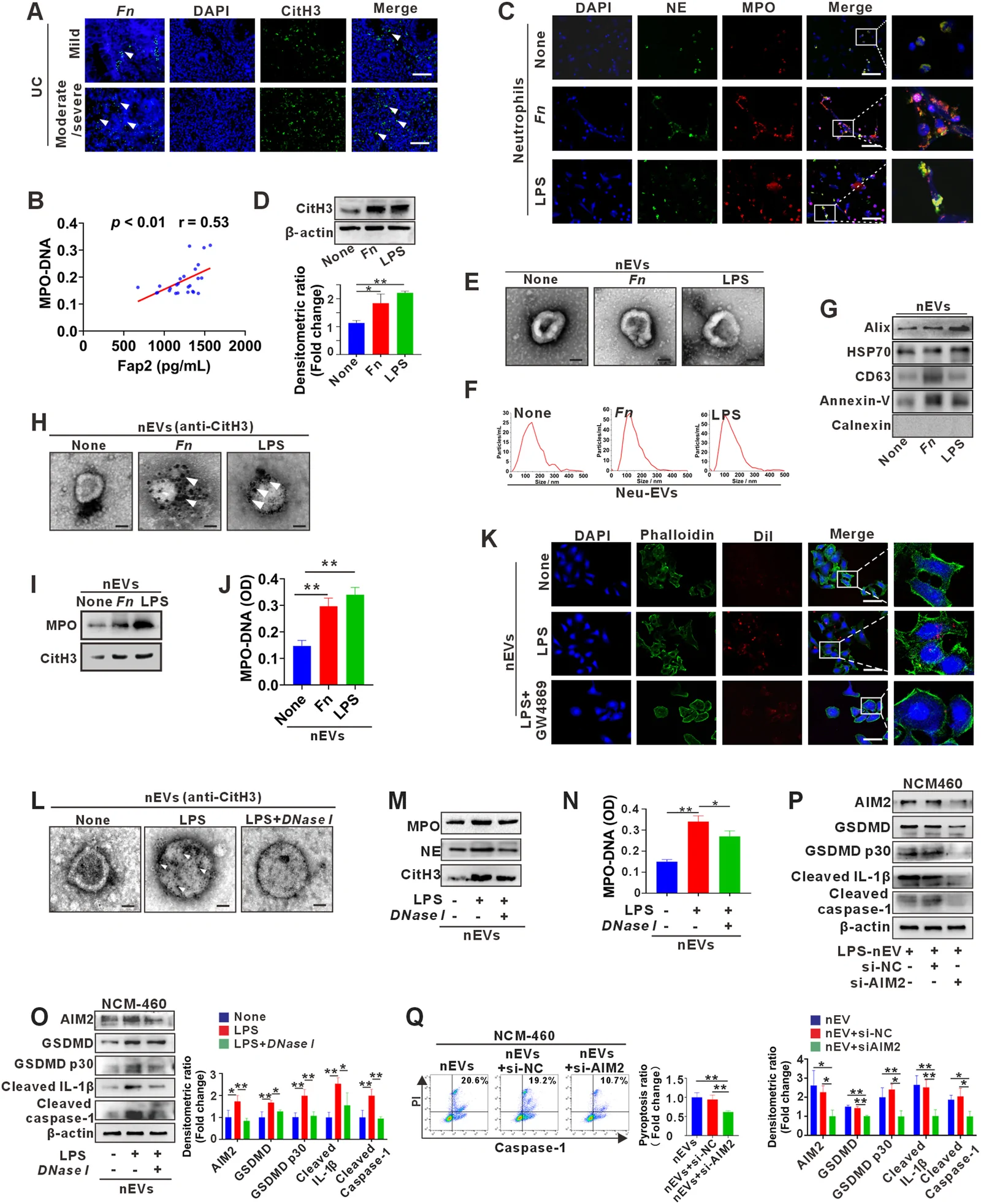
Neutrophil extracellular traps-laden EVs from *Fn*-stimulated neutrophils are linked to AIM2-dependent pyroptosis in intestinal epithelial cells. **(A)** Representative images of fluorescence *in situ* hybridization detection for *Fn* and immunofluorescence images of CitH3 from patients with ulcerative colitis. **(B)** Correlation between serum levels of Fap2 and MPO-DNA of patients with ulcerative colitis. **(C)** Representative immunofluorescence images for MPO and NE in neutrophils treated with *Fn* at a multiplicity of infection (MOI) of 100:1 or LPS (2 μg/mL) for 4 h. **(D)** Western blotting analysis of CitH3 expression in neutrophils treated with *Fn* at a MOI of 100:1 or LPS (2 μg/mL) for 4 h CitH3 protein levels were quantified by densitometry and normalized to β-actin and are shown as fold changes relative to the control (bottom panel). **(E)** Representative TEM micrographs of nEVs with various treatments for 20 h. **(F)** Nanoparticle tracking analysis of nEVs with various treatments for 20 h. Distribution of nEV particle concentration (*y* axis) by size (*x* axis). **(G)** Western blotting analysis of Alix, HSP70, CD63, Annexin-V, and Calnexin in nEVs from neutrophils stimulated with or without *Fn* at a MOI of 100:1 and LPS (2 μg/mL) for 20 h. **(H)** Representative TEM image of nEVs with various treatments for 20 h and then treated with immunogold-labeled anti-CitH3 antibodies. The arrowheads indicate 5 nm gold particles in nEVs. **(I)** Western blotting analysis of MPO and CitH3 in nEVs from neutrophils treated with or without *Fn* at a MOI of 100:1 and LPS (2 μg/mL) for 20 h. **(J)** ELISA analysis for MPO-DNA in nEVs from neutrophils treated with or without *Fn* at a MOI of 100:1 and LPS (2 μg/mL) for 20 h. **(K)** Representative immunofluorescence images of DAPI (blue) and Phalloidin (green), and Dil (indicating nEVs) in NCM-460. **(L)** Representative TEM image of nEVs from conditioned medium of neutrophils treated with LPS or LPS + *DNase I* for 20 h, which were immunogold-labeled with anti-CitH3 antibodies. The arrowheads indicate 5 nm gold particles in nEVs. **(M)** Western blotting analysis of MPO, NE, and CitH3 in nEVs from neutrophils treated with LPS (2 μg/mL) or LPS + *DNase I* (100 U/mL) for 20 h. **(N)** ELISA analysis for MPO-DNA in nEVs from neutrophils treated with LPS or LPS + *DNase I* for 20 h. **(O)** Western blotting analysis of AIM2, GSDMD, GSDMD p30, cleaved IL-1β, and cleaved caspase-1 in NCM-460 treated with various nEVs for 24 h. The protein expression was quantified by densitometry and normalized to β-actin and is shown as fold changes relative to the LPS group (right panel). **(P)** Western blotting analysis of AIM2, GSDMD, GSDMD p30, cleaved IL-1β, and cleaved caspase-1 in NCM-460 (pretreated by si-AIM2 or si-NC) treated with nEVs from neutrophils treated with LPS for 24 h. The protein expression was quantified by densitometry and normalized to β-actin and was shown as fold changes relative to the si-AIM2 group (bottom panel). **(Q)** Propidium iodide and active caspase-1 double staining of pyroptotic cell death by flow cytometry in NCM-460 with the same treatment as in Figure 4P. All data analyzed were shown as mean ± standard deviation in three independent experiments. One-way ANOVA followed by Newman–Keuls’ multiple comparison test was used to determine the significance of the differences among the three groups. ∗*P* < 0.05 and ∗∗*P* < 0.01. White scale bars: 200 μm. Black scale bars: 50 nm. CitH3, citrullinated modification of histone 3; *Fn*, *Fusobacterium nucleatum*; MPO, myeloperoxidase; NE, neutrophil elastase; nEV, neutrophil extracellular vesicle; TEM, transmission electron microscopy; DAPI, 4′’,6-diamidino-2-phenylindole; *DNase I*, *Deoxyribonuclease I*; LPS, lipopolysaccharide.

Activated neutrophils generate NETs via two pathways: suicidal NETosis, a cell death-dependent process triggered by stimuli such as immune complexes, autoantibodies, crystals, or PMA; and vital NETosis, a vesicle-dependent, non-cell death pathway induced by pathogens like *S. aureus* or LPS.^33^ During vital NETosis, nEVs carry NETs composed of double-stranded DNA (dsDNA) and NET-associated proteins, including NE, CitH3, and MPO.^34^ Thus, we hypothesized that AIM2 inflammasome and downstream pyroptosis in NCM-460 cells are related to nEVs-NETs. Then, we isolated nEVs from LPS-stimulated neutrophils with or without *DNase I* for subsequent experiments. Transmission electron microscopy, nanoparticle tracking analysis, and marker detection confirmed the presence of isolated nEVs (Fig. S4A–S4C). Immunofluorescence showed that nEVs, particularly those induced by LPS, could enter IECs, whereas this process was reduced by the vesicle inhibitor GW4869 (Fig. 4K). We analyzed whether these nEVs contained NET-related components. Immunoelectron microscopy detected gold-labelled CitH3 in LPS-stimulated nEVs, while Western blotting and ELISA confirmed the presence of NET-related proteins (MPO, NE, and CitH3) and MPO-DNA (Fig. 4L–N). All these NET components in nEVs were partially diminished by *DNase I* (Fig. 4L–N). Western blotting showed that LPS-stimulated nEVs up-regulated AIM2 and downstream pyroptosis-related proteins in NCM-460 cells, which were markedly reduced by *DNase I* (Fig. 4O). AIM2-siRNA effectively knocked down AIM2 expression (Fig. S4D and S4E) and attenuated both the up-regulation of pyroptosis-related proteins and the percentage of pyroptotic cells induced by LPS-stimulated nEVs (Fig. 4P, Q). To further confirm that NETs exert their effects on NCM-460 cells via nEV internalization, we observed that direct NET (PMA stimulation) treatment did not significantly affect AIM2 or downstream pyroptosis proteins (Fig. S4F and S4G), whereas in a co-culture system of NCM-460 and neutrophils (Fig. S4H), inhibiting vesicle formation by GW4869 (Fig. S4I and S4J) or degrading NETs by *DNase I* (Fig. S4K, S4L) from nEVs effectively suppressed AIM2-dependent pyroptosis.

### Blocking the nEVs-NETs/AIM2 inflammasome cascade alleviates UC severity

We established a 3% DSS-induced colitis mouse model with intraperitoneal administration of *Fn*-induced nEVs, nEVs pretreated with *DNase I (Fn*-nEV + *DNase I)*, or A151 to investigate the effect of nEVs-NETs-induced AIM2 inflammasome-dependent pyroptosis on UC severity (Fig. 5A). Mice receiving *Fn*-nEVs exhibited exacerbated weight loss, elevated disease activity index, and pronounced colon shortening (Fig. 5B–E). These effects were significantly attenuated in the *Fn*-nEVs + *DNase I* group (Fig. 5B–E). A151 treatment also partially restored body weight, disease activity index, and colon length (Fig. 5B–E). Serum levels of TNF-α, IL-6, and IL-1β were markedly elevated in mice receiving *Fn*-nEVs but reduced by *DNase I* (Fig. 5F–H). The treatment of A151 also reduced serum levels of TNF-α, IL-6, and IL-1β (Fig. 5F–H). Hematoxylin-eosin staining revealed that destruction of the structural integrity in the colonic mucosal tissue was more severe in the *Fn*-nEVs group, which was partially ameliorated in the *Fn*-nEVs + *DNase I* group (Fig. 5I and J). The treatment of A151 also dramatically reduced DSS-induced intestinal damage and restored the structural integrity of the colonic mucosal tissue (Fig. 5I, J). The spleen indices exhibited similar tendencies in these groups (Fig. 5K). As expected, expression of AIM2, IL-1β, and GSDMD in colon tissues was substantially elevated in the *Fn*-nEVs group but restored in the *Fn*-nEVs + *DNase I* group. IL-1β and GSDMD were also decreased in the A151 treatment group (Fig. 5I). Consistent with previous findings, double immunofluorescence staining confirmed IEC-specific enrichment of AIM2 in *Fn*-nEVs mice, which was abolished by *DNase I* pretreatment (Fig. 5L).

**Figure 5:**
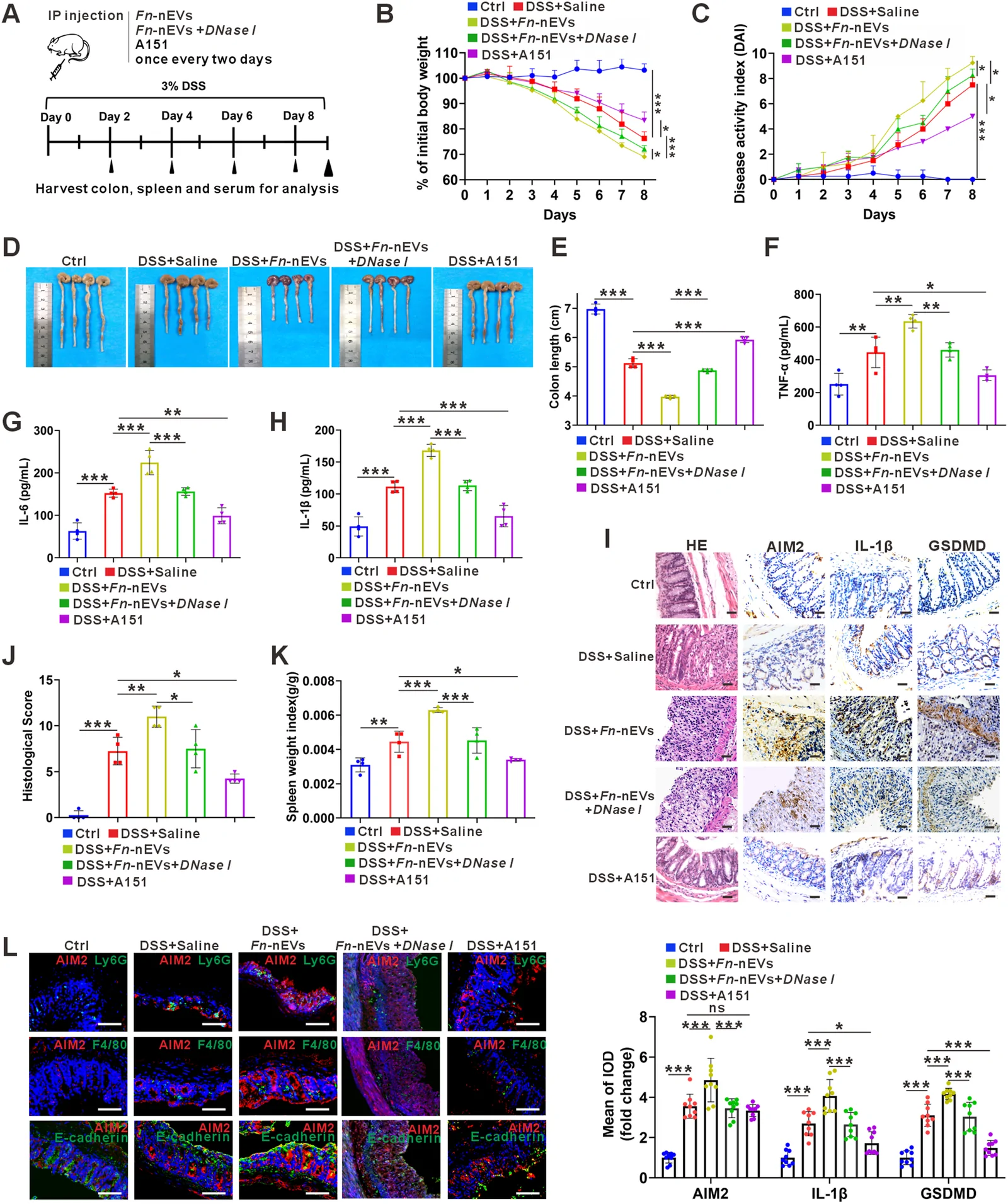
Blocking the nEVs-NETs/AIM2 inflammasome cascade alleviates ulcerative colitis severity. **(A)** Schematic diagram of the study. The mouse model was induced by continuous 3% dextran sulfate sodium (DSS) feeding for 8 days with *Fn*-nEVs, *Fn*-nEVs pretreated with *DNase I*, or A151 once every two days. **(B)** The body weight of mice was expressed as a percentage of the initial body weight. **(C)** The disease activity index was measured daily. **(D, E)** Representative pictures for the length of the colon and statistical analysis in each group. **(F–H)** ELISA analysis of serum levels of TNF-α (F), IL-6 (G), and IL-1β (H) in each group of mice (*n* = 4). **(I)** Representative colon histology of hematoxylin-eosin staining and immunohistochemistry staining for AIM2, IL-1β, and GSDMD from the 8-day 3% DSS-treated mouse model. The mean of integrated optical density (IOD) for immunohistochemistry-stained materials was analyzed utilizing the Image Pro Plus software, and data from histomorphometric analysis were calculated from 3 random fields of each tissue section from 3 randomly selected mice in each group. **(J)** The histological score of mice in each group. **(K)** The spleen index was measured after sacrifice. **(L)** Representative immunofluorescence images of AIM2 (red) and Ly6G (top), F4/80 (middle), or E-cadherin (bottom) (green) from colon tissues of 3% DSS-treated mice. All data analyzed were shown as mean ± standard deviation. One-way ANOVA followed by Newman–Keuls’ multiple comparison test was used to determine the significance of the differences among these groups. ns, not significant; ∗*P* < 0.05, ∗∗*P* < 0.01, and ∗∗∗*P* < 0.001. Black scale bars: 100 μm; White scale bars: 200 μm. *Fn*-nEVs, *Fn*-stimulated neutrophil extracellular vesicles; NETs, neutrophil extracellular traps; *DNase I*, *Deoxyribonuclease I*; IP, intraperitoneal injection.

## Discussion

UC is an inflammatory bowel disease with complex pathogenesis, but the underlying molecular mechanism is incompletely understood.^4^^,^^35^ Either elevated NETs or the occurrence of pyroptosis has been previously implicated in UC.^36^^,^^37^ However, it remains unknown whether the two immune responses can communicate with each other and how they impact the pathological process of UC. Here, we demonstrated that nEVs carrying the NETs triggered by *Fn* were internalized by IECs to trigger AIM2 inflammasome-dependent pyroptosis, eventually aggravating colitis. Additionally, targeting the nEVs-NETs/AIM2 inflammasome cascade relieved colonic inflammation (Fig. 6).

**Figure 6:**
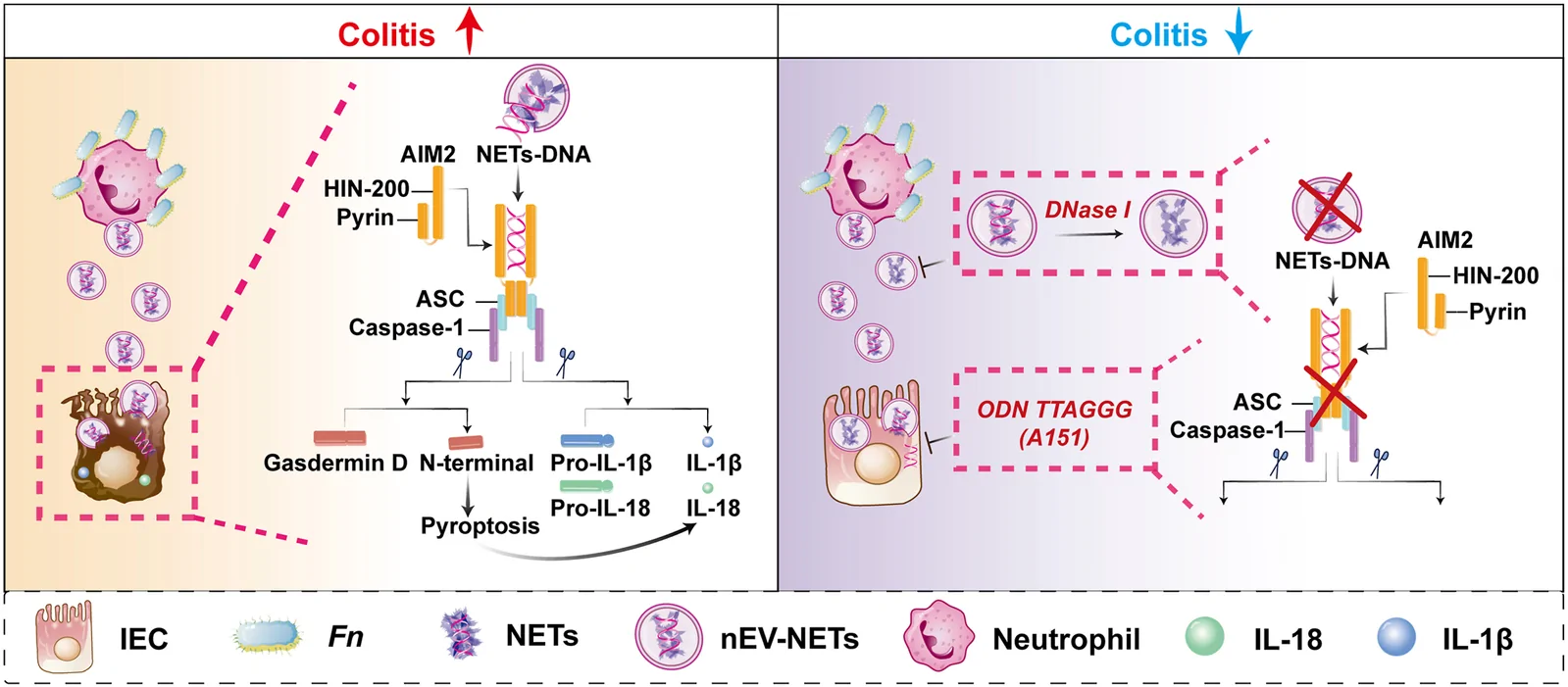
A working model illustrating that nEVs laden with *Fn*-induced neutrophil extracellular traps (NETs) are internalized by intestinal epithelial cells (IECs), inducing AIM2 inflammasome-dependent pyroptosis and thereby exacerbating colitis. Notably, the nEVs-NETs/AIM2 axis represents a promising therapeutic target for ulcerative colitis therapy. *Fn*, *Fusobacterium nucleatum*; nEV, neutrophil extracellular vesicle; *DNase I*, *Deoxyribonuclease I*.

Some hypotheses have proposed that intestinal mucosa damage was related to dysregulated innate immune response in UC.^38^ Therefore, deciphering the regulatory circuits that govern aberrant immune responses in the intestine is essential to understanding the pathophysiology of UC. Pyroptosis leads to the infection of enteropathogenic bacteria and modulates mucosal innate immunity, which play vital roles in inflammatory diseases, including UC.^39^ Most prior studies on pyroptosis in UC have focused on NLRP3 inflammasome-dependent pyroptosis.^40^^,^^41^ Recently, AIM2 inflammasome-dependent pyroptosis in UC has gradually begun to receive attention due to the up-regulation of the AIM2 inflammasome in inflammatory bowel disease. Although one previous study showed that AIM2^−/−^ mice exhibited heightened susceptibility to DSS-induced colitis, this work approached AIM2 solely from a genetic perspective, neglecting its cell-specific expression and the systemic effects of its downstream effectors.^42^ We noticed this point and specifically studied the type of cells undergoing AIM2 inflammasome-dependent pyroptosis in mouse and human tissue samples. We demonstrated that AIM2 inflammasome-dependent pyroptosis occurred in IECs and exacerbated colitis in a DSS-induced murine model. These findings significantly advance our understanding of the AIM2 inflammasome in UC pathogenesis.

Neutrophils, the most abundant immune cells in human circulation, serve as the first line of defense against infection and inflammation by rapidly migrating to sites of tissue injury or pathogen invasion.^43^ NETs are DNA scaffolds coated with antimicrobial granule proteins that trap and neutralize pathogens, thereby mediating antibacterial immune responses.^44^ Prior work identified elevated levels of neutrophil- and NET-associated proteins in UC biopsy tissue.^45^ Consistent with these findings, we observed elevated NET levels in UC patients and a DSS-induced murine colitis model. Notably, NET levels positively correlated with UC disease severity. Excessive NET production can injure the intestinal epithelium and affect the function of the intestinal mucosal barrier, but the mechanism is currently unclear.^19^^,^^46^ Emerging evidence suggests NETs drive pyroptosis to exacerbate pathologies such as sepsis and traumatic brain injury.^20^^,^^47^ We therefore hypothesized that NETs could promote UC progression by inducing pyroptosis, leading to IL-1β and IL-18 release and sustained inflammation.

The occurrence of pyroptosis depends on the assembly of inflammasomes.^48^ Prior studies demonstrate that NETs activate the AIM2 inflammasome, promoting alveolar macrophage pyroptosis.^22^ AIM2 directly detects and binds to cytosolic DNA, positioning it as a critical innate immune sensor.^49^^,^^50^ NETs, which consist of DNA scaffolds decorated with granular proteins,^51^ are recognized by AIM2 via their dsDNA content. The present study indicated that AIM2 inflammasome-dependent pyroptosis markers correlated positively with NET levels, underscoring their mechanistic interplay in UC. However, other key DNA sensors, including Toll-like receptor 9 (TLR9) and cyclic GMP-AMP synthase (cGAS),^52^ may contribute to UC pathogenesis. TLR9–ERK signaling activation^53^ and elevated cGAS expression in active UC^54^ highlight additional pathways requiring exploration. Whether NETs engage in crosstalk with TLR9 or cGAS pathways to drive UC progression remains an open question.

IECs, critical components of the intestinal barrier, fight against pathogenic bacteria through cell pyroptosis. Structural weakening of the intestinal mucosal barrier due to IEC dysregulated cell death represents an early pathogenic event in UC.^41^^,^^55^ Coculture experiments showed that AIM2 inflammasome-dependent pyroptosis in IECs was related to NETs. Given the non-phagocytic nature of IECs, we hypothesized that NET-induced pyroptosis involved EVs, key mediators of intercellular crosstalk.^56^ Mounting evidence demonstrates that EVs harbor molecules regulating inflammation and immune responses, playing pivotal roles in inflammatory cascades.^57^ Vital NET formation, induced by stimuli such as *S. aureus* or LPS, involves NE- and MPO-mediated chromatin decondensation, packaging into nuclear-derived vesicles, and their release from intact neutrophils to form NETs locally.^33^ During vital NETosis, the isolated EVs contain NETs composed of dsDNA and a multitude of NET-associated proteins, such as NE, CitH3, and MPO.^34^ In this present study, a coculture system (neu-LPS-GW4869 with NCM-460 cells) confirmed that NET-induced AIM2 inflammasome activation depends on nEVs. Based on vital NETosis mechanisms, we propose that LPS-activated neutrophils release NETs, which are packaged into nEVs for IEC delivery, triggering AIM2 inflammasome-dependent pyroptosis. Here, immunoelectron microscopy, western blotting, and ELISA confirmed that nEVs carried key NET components: CitH3, NE, MPO, and MPO–DNA complexes. Moreover, the isolated LPS-induced nEVs were proven to be capable of entering IECs to induce AIM2 inflammasome-dependent pyroptosis, which indirectly confirmed our hypothesis.

Although prior research and the current study revealed that NETs-DNA could activate the AIM2 inflammasome.^22^^,^^23^ The mechanism underlying the binding of NETs-DNA to AIM2 remains unclear. Biochemical studies^58^^,^^59^ have shown that AIM2 can directly bind to DNA, with three major features: i) the interaction is HIN domain-dependent, as the HIN domain has two oligonucleotide/oligosaccharide binding folds that have high affinity to DNA; ii) it is independent of DNA sequence, GC content, or origin but the DNA length must be at least 80 base pairs; i) there is a preference for dsDNA. NETs comprise a complex mixture of components, and the specific sequences within NETs-DNA that directly interact with AIM2, as well as the defining features of these fragments, remain to be elucidated and warrant further investigation.

Prior studies have established dysbiosis in the gut microbiota of UC patients.^60^ UC patients are at a high risk of developing colorectal cancer, one of the most serious complications of UC.^61^ *Fn*, which was demonstrated to be enriched in colorectal cancer and to promote colorectal cancer progression via neutrophil extracellular traps (NETs) in our previous research,^24^ has also been implicated as a key pathogen in UC.^25^^,^^30^ Based on these observations, we investigated the association between *Fn* abundance and NET levels in UC patients. Here, human UC biopsy tissue analyses and *in vitro* models revealed that elevated NET levels correlated with *Fn* enrichment in UC. These findings advance our understanding of UC pathogenesis by linking microbiota-driven immune dysregulation to NET-mediated inflammation. Extending these observations, our study demonstrates that *Fn* actively stimulates neutrophils to release extracellular vesicles laden with NETs. These vesicles are internalized by IECs, triggering AIM2 inflammasome activation and subsequent pyroptotic cell death. This pathway provides a direct mechanistic connection between microbiota dysbiosis and epithelial injury, positioning *Fn*-induced NETs-laden vesicles as key amplifiers of mucosal inflammation. Furthermore, recent studies have highlighted additional mechanisms by which *Fn* exacerbates UC. Wei et al reported that *Fn*-derived EVs promoted experimental colitis severity through miR-574-5p/CARD3-dependent autophagy activation, leading to epithelial barrier dysfunction and increased inflammation.^62^ Additionally, Boonyaleka et al demonstrated that *Fn* infection activated the noncanonical inflammasome, enhancing IL-1α secretion and exacerbating inflammatory responses in DSS-induced colitis models.^63^ Collectively, these findings define a microbiota–immune–epithelial axis in UC, in which the release of *Fn*-driven NETs not only marks neutrophil activation but also exacerbates disease via AIM2 inflammasome-dependent epithelial damage. The engagement of multiple inflammasome pathways by *Fn* may further amplify intestinal inflammation and epithelial cell death, underscoring its multifaceted role in UC pathogenesis and highlighting potential targets for therapeutic intervention.

Current UC therapies comprise non-targeted approaches and biologic agents. Although biologic therapies are effective in many patients, the response to initial treatment is lost over time in up to 50% of patients.^64^ Thus, identifying novel therapeutic targets for UC is critically needed. In this study, we investigated UC pathogenesis and aimed to identify immune cell interaction-based therapeutic targets. *In vivo* experiments showed that degrading nEVs-NETs by *DNase I* or inhibiting AIM2 inflammasome activation by A151 attenuated pyroptosis and colitis severity. These results position the nEVs-NETs/AIM2 axis as a promising therapeutic target for UC. Although mouse models have verified the effectiveness of the above treatment, the pathogenesis of UC in humans involves complex genetic and environmental interplay. Future clinical trials are essential for translational validation.

There also exist some limitations in the study. Firstly, larger sample sizes in UC are required to validate these findings in future studies. Secondly, we analyzed NETs holistically to assess their role in AIM2 inflammasome-dependent pyroptosis in IECs. Future work should identify whether specific NETs-associated DNA fragments drive AIM2 inflammasome activation. Finally, the complex pathogenesis of UC warrants validation using *Fn* isolates derived from clinical UC patients, as well as in chronic colitis murine models, to strengthen the potential for clinical translation of these findings.

In summary, this study investigated the roles of NETs and AIM2 inflammasome-dependent pyroptosis, established their clinical correlation with UC severity, and elucidated their interplay in driving UC pathogenesis. We revealed that nEVs laden with NETs formed in response to abundant *Fn* stimulation were internalized by IECs, triggering AIM2 inflammasome-dependent pyroptosis and thereby exacerbating colitis. Notably, the nEVs-NETs/AIM2 axis represents a promising therapeutic target for UC therapy.

## CRediT authorship contribution statement

**Linwei Xiang:** Writing – original draft. **Yuqing Zhao:** Data curation. **Yaqian Duan:** Data curation. **Jia Xu:** Methodology. **Yan You:** Methodology. **Xuehua Kong:** Data curation. **Jianbo Zhang:** Data curation. **Rui Wu:** Methodology, Funding acquisition. **Lan Zhou:** Project administration, Conceptualization. **Liang Duan:** Writing – original draft, Supervision, Project administration, Funding acquisition, Conceptualization.

## Ethics declaration

All experiments involving human samples were conducted according to the ethical policies and procedures approved by the Ethical Committee of the First Hospital affiliated with Chongqing Medical University (Approval no. K2023-305). All experiments involving animals were conducted according to the ethical policies and procedures approved by the Animal Ethics Committee of Chongqing Medical University (Approval no. 2022141).

## Data availability

All data generated or analyzed during this study are included in this published article and the Supplementary material.

## Funding

This work was supported by the 10.13039/501100001809National Natural Science Foundation of China (No. 82472354), 10.13039/501100005230Natural Science Foundation of Chongqing, China (No. CSTB2023NSCQMSX0037, CSTB2024NSCQ-MSX0342), Chongqing medical scientific research project (Joint project of Chongqing Health Commission and Science and Technology Bureau) (China) (No. 2025ZDXM016), and Senior Medical Talents Program of Chongqing for Young and Middle-aged (China) (No. 2022-15).

## Conflict of interests

The authors declared no conflict of interests.
