# Supplementary material for: Activation of AIM2 inflammasome cascade by extracellular trap-laden neutrophil vesicles triggered by Fusobacterium nucleatum exacerbates ulcerative colitis
Source: Genes Dis. 2025 Dec 24;14(2):102004. doi: 10.1016/j.gendis.2025.102004 (PMC13684999; doi:10.1016/j.gendis.2025.102004)
Supplement: Supplementary file 1 [file mmc1.docx]

**Supplementary Information**

Figure S1. (**A)** The body weight of mice was expressed as a percentage of the initial body weight. (**B)** Disease Activity Index (DAI), calculated daily based on body weight loss, stool consistency, and presence of fecal/anal blood. (**C)** Spleen index (spleen-to-body weight ratio) measured post-euthanasia. (**D)** The histological score of mice in each group. The statistical results are presented as mean±SD. Differences between Ctrl and DSS groups were tested by Student’s t-test. ***p* < 0.01, ****p* < 0.001.

Abbreviations: Ctrl, control; DSS, dextran sulfate sodium.

Figure S2. **(A)** Representative colon histology of H&E, IF images of CitH3 and IHC staining for AIM2, IL-1β and GSDMD from the 8-day 3% DSS-treated mouse model. **(B-E)** ELISA analysis for MPO-DNA (B), AIM2 (C), GSDMD (D), and IL-18 (E) levels in the serum from mouse models with mild UC (n=11) and moderate/severe UC (n=9). (**F-H)** Correlation between serum levels of MPO-DNA and AIM2 (F), GSDMD (G) or IL-18 (H) in DSS-induced UC mouse models. The statistical results are presented as median (IQR). The Mann-Whitney test was used to determine the significance of the differences between the two groups. **p* < 0.05, ***p* < 0.01, ****p* < 0.001. Black scale bars: 100 μm; White scale bars: 200 μm. Abbreviations: CitH3, citrullinated modification of histone 3; IHC, immunohistochemistry; DSS, dextran sulfate sodium; IF, immunofluorescence; IQR, interquartile range.

Figure S3. (**A)** Representative IF images for MPO and NE in neutrophils treated with *E.coli* at multiplicity of infection (MOI) of 100:1 for 4 h. (**B)** Western blot analysis of CitH3 expression in neutrophils treated with *E. coli* at multiplicity of infection (MOI) of 100:1 or LPS (2 μg/mL) for 4 h. CitH3 protein levels were quantified by densitometry and normalized to β-actin and are shown as fold changes relative to the control (right panel). Data are shown as means±SD in three independent experiments. Oneway ANOVA followed by Newman-Keuls’ multiple comparison test was used to determine the significance of the differences among the three groups. ns, not signiﬁcant, ***p* < 0.01. White scale bars: 200 μm. Abbreviations: IF, immunofluorescence; MPO, myeloperoxidase; NE, neutrophil elastase; *E. coli*, *Escherichia coli*; CitH3, citrullinated modification of histone 3; LPS, lipopolysaccharide;

Figure S4. (**A)** Representative TEM images of nEVs. (**B)** NTA of EVs isolated from neutrophils. Distribution of EV particle concentration (y axis) by size (x axis). (**C)** Western blot analysis of Alix, HSP70, CD63, Annexin-V and calnexin in EVs from neutrophils treated with 2 μg/mL LPS or 2 μg/mL LPS and 100 U/mL *DNase Ⅰ* for 20 h. (**D)** qRT-PCR analysis for the mRNA levels of AIM2 in NCM460 treated with si-AIM2-1, si-AIM2-2, or si-AIM2-3. (**E)** Western blot analysis of AIM2 expression in NCM460 of (D). AIM2 protein levels were quantified by using densitometry and normalized to β-actin and are shown as fold changes compared to the control (right). Data are shown as means±SD in three independent experiments. (**F)** Western blot analysis of AIM2, GSDMD, GSDMD p30, cleaved IL-1β, and cleaved caspase-1 in NCM-460 treated with 2 µg/ml NETs for 24 h. The protein expression was quantified by densitometry and normalized to β-actin and is shown as fold changes relative to the ctrl group (right panel). Data are shown as means±SD in three independent experiments. (**G)** Flow cytometry analysis of pyroptotic NCM-460 cells (from panel A) using PI and active caspase-1 double staining. Data represent three independent experiments. Data are shown as means±SD in three independent experiments. (**H)** Procedure for pyroptosis analysis by co-culture of neutrophils with different conditions and NCM-460 for 48 h. **(I)** Western blot analysis of AIM2, GSDMD, GSDMD p30, cleaved IL-1β and cleaved caspase-1 in NCM-460 after co-cultivation with neutrophils treated with LPS (1 μg/mL) or LPS+GW4869 (2 μg/mL) for 48 h. The protein expression was quantified by densitometry and normalized to β-actin and is shown as fold changes relative to the LPS group (right panel). **(J)** PI and active caspase-1 double staining of pyroptotic cell death by flow cytometry in NCM-460 with same treatment as I. **(K)** Western blot analysis of AIM2, GSDMD, GSDMD p30, cleaved IL-1β, and cleaved caspase-1 in NCM-460 cells after co-cultivation with neutrophils treated with LPS (1 μg/mL) or LPS+*DNase I* (100 U/mL) for 48 h. The protein expression was quantified by densitometry and normalized to β-actin and is shown as fold changes relative to the LPS group (right panel). **(L)** PI and active caspase-1 double staining of pyroptotic cell death by flow cytometry in NCM-460 cells with the same treatment as K. The Student’s t-test was used to determine the significance of the differences between the two groups. Oneway ANOVA followed by Newman-Keuls’ multiple comparison test was used to determine the significance of the differences among the three groups. ns, not signiﬁcant; **p* < 0.05, ***p* < 0.01, ****p* < 0.001. Black scale bars: 50 nm. Abbreviations: *DNase I*, *Deoxyribonuclease* *I*; nEV, neutrophil extracellular vesicle; HSP70, heatshockprotein 70; LPS, lipopolysaccharide; NETs, neutrophil extracellular traps; Neu, neutrophil; NTA, nanoparticle tracking analysis; PI, propidium iodide; TEM, transmission electron microscopy.

Table S1: sequences of siRNA and RT-qPCR primer

| Name | Sequences (5ʹ→3ʹ) |
| --- | --- |
| si-RNA negative control | F: 5ʹ-UUCUCCGAACGUGUCACGUTT-3ʹ  R: 5ʹ-ACGUGACACGUUCGGAGAATT-3ʹ |
| si-AIM2-1 | F: 5ʹ-CCAGCAGGAAUCUAUCAGATT-3ʹ  R: 5ʹ-UCUGAUAGAUUCCUGCUGGTT-3ʹ |
| si-AIM2-2 | F: 5ʹ-GCAAGCAGGAGAUGUUUCATT-3ʹ  R: 5ʹ-UGAAACAUCUCCUGCUUGCTT-3ʹ |
| si-AIM2-3 | F: 5ʹ-GGAAGGAGAUAAGGUUCGATT-3ʹ  R: 5ʹ-UCGAACCUUAUCUCCUUCCTT-3ʹ |
| AIM2 | F: 5ʹ- AAAGCTGGTGAAACCCCGAA-3ʹ  R: 5ʹ-GGCCTTAATAACCTTTATGGTGCT-3ʹ |
| GAPDH | F: 5ʹ- CAGCGACACCCACTCCTC-3ʹ  R: 5ʹ- TGAGGTCCACCACCCTGT-3ʹ |

Abbreviations: AIM2, absent in melanoma 2; F, forward Primer; GAPDH, lyceraldehyde-3-phosphate dehydrogenase; R, reverse Prime.
